# Protective effects of total flavonoids from Flos Puerariae on retinal neuronal damage in diabetic mice

**Published:** 2013-09-20

**Authors:** Dai Li, Fang Yang, Hongke Cheng, Chao Liu, Ming Sun, Kaili Wu, Ming Ai

**Affiliations:** 1 Department of Ophthalmology, Renmin Hospital of Wuhan University, Wuhan, China; 2 Department of Ophthalmology, Hubei University of Science and Technology, Xianning, China; 3 Department of Ophthalmology, Hubei University of Medicine Affiliated People’s Hospital of Shiyan, Shiyan, China; 4 Hubei Province Key Laboratory on Cardiovascular, Cerebrovascular, and Metabolic Disorders, Hubei University of Science and Technology, Xianning, China; 5 State Key Laboratory of Ophthalmology, Zhongshan Ophthalmic Center, Sun Yat-sen University, Guangzhou, China; 6 Wuhan Aier Eye Hospital, Wuhan, China

## Abstract

**Purpose:**

To investigate the potential protective effects of total flavonoids from Flos Puerariae (TFF) on retinal neural cells in diabetic mice.

**Methods:**

C57BL/6J mice were intraperitoneally injected with streptozotocin to generate type I diabetes in a murine model, as indicated by blood glucose levels ≥11.1 mmol/l. TFF was administered intragastrically at a dose of 50, 100, or 200 mg/kg/day. After 10 weeks of administration, the mice were euthanized, and the eyes were dissected. Retinal histology was examined, and the thickness of the retina was measured. Ultrastructural changes in the retinal ganglion cells and capillary basement membrane were observed with electron microscopy. Apoptosis of retinal neural cells was determined with the terminal deoxynucleotidyl transferase-mediated dUTP-biotin nick end-labeling assay. Bax and Bcl-2 expression in the retinal tissues was determined with immunohistochemical staining and western blotting.

**Results:**

Compared with the diabetic mice, the blood glucose level decreased (p<0.01) and the bodyweight increased (p<0.05) in the 100 and 200 mg/kg TFF-treated groups. The thickness of the retina significantly increased (p<0.01), and the retinal capillary basement membrane (BM) thickness was reduced in the 100 and 200 mg/kg TFF-treated diabetic mice (DM). The 100 and 200 mg/kg TFF treatments also attenuated the diabetes-induced apoptosis of retinal neural cells. Consistent with these effects, TFF treatment decreased the Bax expression level and, concurrently, increased the ratio of Bcl-2 to Bax.

**Conclusions:**

TFF attenuated diabetes-induced apoptosis in retinal neurons by inhibiting Bax expression and increasing the ratio of Bcl-2 to Bax, which suggests that TFF might prevent retinal neuronal damage in diabetes mellitus.

## Introduction

Diabetic retinopathy (DR) is the most common complication of diabetes mellitus and one of the major causes of vision loss across the globe. Almost all patients with diabetes suffer different degrees of retinopathy after 20 years’ duration of diabetes [1]. It has long been believed that DR is a microvascular disease [2]. In recent years, an increasing body of evidence has suggested that neuronal cell death of the retina is a critical component of DR [3-7]. With microvascular lesions, neurodegeneration might occur in the early stage of diabetic retinopathy, preceding retinal vascular complications in humans and experimental animals [8,9]. Abu El-Asrar et al. reported that ganglion cells in diabetic retinas express several proapoptotic molecules, such as caspase-3, Fas, and Bax, suggesting that these cells are the most vulnerable population in diabetic retinopathy [9]. Another study showed that neurotoxicity causes permanent impairment of visual function due to cell death of the inner retinal and ganglion cells [10]. Intervention in the apoptosis of ganglion cells may allow us to therapeutically delay or ameliorate neural cell loss in retinal neurodegenerative conditions related to diabetes.

Traditional Chinese medicine has been widely used for centuries and can play a unique therapeutic role in treating many human diseases. Flos Puerariae, a well known Chinese medicine compound, is the dry bud of *Pueraria lobata* (a plant in the genus Pueraria in the pea family Fabaceae, subfamily Faboideae). Seven isoflavones have been identified in the Flos Puerariae extract, including four isoflavone glycosides (tectoridin, tectorigenin7-oxyloglucoside, 6-hydroxy-genistein-6,7-diglucoside, and glycitin) and three aglycones (tectorigenin, glycitein, and genistein). Three saponins (soyasaponin I, kaikasaponin III, and kakkasaponin I) have been identified in the extract [11-13]. The extract of Flos Puerariae has been reported to have a wide range of pharmacological effects, including prevention of the effects of excessive alcohol ingestion (hangovers) [14], antioxidant action [15], a liver protective effect [16], an estrogenic-like effect [17], hypolipidemic and hypoglycemic effects [18,19], and an antiapoptotic effect [20,21]. Considering the close correlation between DR and the apoptosis of retinal neural cells, total flavonoids from Flos Puerariae (TFF) may improve DR by hypoglycemia and inhibiting neuronal damage.

To test this hypothesis, we examined whether TFF has a neuroprotective effect on retinal neural cells in the streptozotocin (STZ)-induced diabetic mouse model. The regulation of various apoptotic-related genes, such as Bax and Bcl-2, has been used to evaluate apoptotic activity in tissues. The balance of Bax and Bcl-2 ensures a steady-state of the cell, and changes in the Bcl-2/Bax expression ratio determine cell survival or apoptosis [21-23]. In this study, we investigated the effect of oral administration of TFF on hyperglycemia and the morphology of the diabetic mouse retina, as well as the effect on apoptosis of the retinal neurons in relation to the expression of apoptotic-related genes (Bax and Bcl-2).

## Methods

### Extract of total flavonoids from Flos Puerariae

In this study, total flavonoids were extracted from Flos Puerariae using an ultrasonic-assisted extraction method as previously reported [24]. Briefly, Flos Puerariae was extracted in 40% methanol and broken down by ultrasound for 4 h. Petroleum ether was blended with the brown viscous extract, and the extract was separated into two layers, a petroleum ether layer (to remove fat-soluble impurities) and a brown water layer. The brown water layer was collected and concentrated with anhydrous ethanol. The dark brown sediment (predominantly polysaccharide) was precipitated. The supernatant ethanol solution was concentrated with anhydrous ethanol. The remaining solution comprised the total flavonoids extracted from Flos Puerariae.

### Experimental animals and treatment

Fifty male C57BL/6J mice, weighing 22±2.0 g, were purchased from the Wuhan University Center for Animal Experimentation. Animals were housed in a specific pathogen-free animal house according to the guidelines established by the Association for Research in Vision and Ophthalmology’s Statement for the Use of Animals in Ophthalmic and Vision Research. The animals were fed by adaptive feeding (with free food and water) for 1 week. They were housed with a 12 h:12 h light-dark cycle at 23–25 °C and 55%–60% humidity.

Forty C57BL/6J mice were intraperitoneally injected with STZ (Sigma Aldrich, St. Louis, MO) to generate the type I diabetic model, as described previously [25,26] with modifications. We induced diabetes in mice with multiple intraperitoneal injections of STZ dissolved in sodium citrate buffer (pH 4.5) at a low-dose level of 50 mg/kg bodyweight daily for 5 consecutive days. Ten age-matched mice were given the same volume of sodium citrate instead of STZ and served as controls. A sample of whole blood was drawn from the tail vein of each mouse by cutting the tail, and blood glucose was measured with a glucometer (Sinocare Inc., Changsha, China) at 2 and 4 weeks post-injection. After 4 weeks of exposure to STZ, 36 mice with blood glucose levels ≥11.1 mmol/l were considered diabetic. They were randomly divided into four groups (n=9, each group): the diabetic group (DM), the 50 mg/kg/day TFF-treated group (DM+TFF I), the 100 mg/kg/day TFF-treated group (DM+TFF II), and the 200 mg/kg/day TFF-treated group (DM+TFF III). The mice in the drug intervention groups had intragastric TFF administration daily. The mice in the DM group did not receive treatment. The mice were weighed weekly, and blood glucose was measured at the end of the course of treatment. After 10 weeks of TFF administration, the mice were euthanized by intraperitoneal injetion of 1% pentobarbital (50 mg/kg), and the eyes were dissected. Four eyes from four mice in each group were immediately fixed in 4% paraformaldehyde solution for histopathological examination. The remaining retinas were carefully dissected under a stereomicroscope. Three retinas from three individuals in each group were immediately placed in a 2.5% glutaraldehyde solution for ultrastructural analysis. The remaining retinas were frozen immediately in liquid nitrogen and stored at −80 °C for western blotting analysis.

### Hematoxylin and eosin staining and morphology examination

Four paraformaldehyde-fixed eye tissues from each group were embedded in parafﬁn and sectioned at 5 µm thickness each for hematoxylin and eosin (H&E) staining, terminal deoxynucleotidyl transferase-mediated dUTP nick end-labeling (TUNEL) analysis, and immunohistochemical staining. All color micrographs were collected using a microscope and digital camera (Axioplan 2 imaging; Carl Zeiss, Inc., Shanghai, China; camera equipped with Spot Software ver. AxioVision Rel 4.8). The morphology of the retina, including measurements of the thickness of the total retina, inner nuclear layer (INL), and outer nuclear layer (ONL), was determined with Spot Software ver. AxioVision Rel 4.8. The thickness was measured on the magnified images (×400) of the retinal equator at six points: three on either side of the retinal equator, located approximately 500 to 600 μm apart from the ora serrata. The mean thickness value of the four eyes was recorded as the representative value for each group. The results of the five groups were analyzed with ANOVA (ANOVA).

### Terminal deoxynucleotidyl transferase-mediated dUTP nick end-labeling assay

The TUNEL assay was performed using the TUNEL Apoptosis Assay Kit (Roche Applied Science, Nutley, NJ) according to the manufacturer’s instructions. Three sections from each group were selected for the TUNEL assay. The dewaxed sections were treated with 20 μg/ml proteinase K for 15 min, incubated with equilibration buffer for 15 s, and incubated with terminal deoxynucleotidyl transferase (TdT) enzyme at 37 °C for 1 h. Antibody blocking proceeded for 5 min, and peroxidase (POD; conjugated with horseradish peroxidase) was incubated with the sections. The sections were incubated with substrate 3,3′-diaminobenzidine for 8 min.

Three visual fields (×400) under microscope (Axioplan 2 imaging) per section were randomly chosen to count the number of TUNEL-positive cell nuclei (apoptotic nuclei) and total cell nuclei in the ganglion cell layer (GCL) and the INL, respectively. The percentage of TUNEL-positive cells, termed the apoptotic index (AI), was calculated using the following formula: AI=apoptotic nuclei/total nuclei ×100%. The mean value of the eyes from four mice was recorded as the representative value for each group. The results of the five groups were statistically analyzed with ANOVA.

### Immunohistochemistry assays of Bax and Bcl-2

To examine Bax and Bcl-2 protein expression in the retinal tissue, immunohistochemical staining was performed on paraffin sections as previously reported [27]. Three sections of each paraffin-embedded eye tissue from each group underwent immunohistochemical staining. Tissue sections were incubated with polyclonal primary antibodies against Bax or Bcl-2 (Santa Cruz Biotechnologies, Santa Cruz, CA) overnight at 4 °C. After being washed with PBS (phosphate buffer saline), the sections were incubated with biotinylated horse anti-mouse immunoglobulin G for 30 min and then incubated with the avidin-biotin-peroxidase complex using an ABC kit. The reaction was visualized by color development with 3, 3′-diaminobenzidine tetrahydrochloride. All sections were counterstained with hematoxylin. Images from the immunohistochemical studies of Bax and Bcl-2 protein expression (×400) were photographed with a microscope and digital camera (Axioplan 2 imaging; the camera was equipped with Spot Software ver. AxioVision Rel 4.8).

### Transmission electron microscopy detection

Three pieces of retina approximately 2 mm × 3 mm in size were isolated from each eyecup, approximately 2 mm from the optic disc, following the protocol described previously [28]. Briefly, retina tissues were immediately preﬁxed in 2.5% glutaraldehyde for 2 h and then ﬁxed in 1% osmium tetroxide. Sequentially, the tissues were dehydrated and embedded in Epon 812. The tissues were sectioned at a thickness of 50–60 nm using an ultramicrotome and double-stained with uranyl acetate and lead citrate. The ultrastructure of the retina tissues was observed using a transmission electron microscope (TEM H-600, Hitachi, Tokyo, Japan).

### Western blot analysis of Bax and Bcl-2

Western blot tests were performed as described previously [29]. The retinas in each group were pooled and homogenized in ice-cold lysis buffer (20 mM Tris, pH 7.5, 150 mM NaCl, 1 mM EDTA, 1 mM EGTA, 1% Triton X-100, 2.5 mM sodium pyrophosphate, 1 mM β-glycerolphosphate, 1 mM Na_3_VO_4_, 1 μg/ml aprotinin leupeptin and pepstatin, and1 mM phenylmethylsulfonyl ﬂuoride) and centrifuged at 12,890 ×*g* for 15 min at 4 °C. The supernatant was collected, and the protein concentration was measured using the bicinchoninic acid protein assay (Beyotime, Jiangsu, China). An equal amount of protein for each sample (60 μg/lane) was separated on 10% sodium dodecyl sulfate–polyacrylamide gel and transferred to a polyvinylidene ﬂuoride membrane. The membranes were blocked in 5% skim milk for 2 h at room temperature before incubation with an antibody against Bax, Bcl-2 (Cell Signaling Technology, Boston, MA), or β-actin (Santa Cruz Biotechnology, Dallas, TX) overnight at 4 °C. The membranes were incubated with horseradish peroxidase-conjugated goat anti-mouse immunoglobulin G (GE Healthcare, Buckinghamshire, UK) for 2 h, and blots were developed using an enhanced chemiluminescence kit (Pierce Biosciences, Rockford, IL). β-actin expression was used as an internal loading control to standardize the amount of loaded protein. Experiments were repeated five times. The intensity of the protein band was semiquantitatively measured with image analysis software (Image-Pro Plus 6.0, Media Cybernetics, Bethesda, MD).

### Statistical analysis

All data were reported as the mean±standard deviation. One-way ANOVA and Student–Newman–Keuls tests were performed to compare the means of the groups using statistical software (SPSS Statistics 17.0, New York, NY). A value of p<0.05 was considered statistically significant.

## Results

### Total flavonoids from Flos Puerariae reduced the blood glucose of diabetic mice

After the full course of exposure to STZ, as shown in Table 1, the blood glucose levels of the diabetic mice significantly increased compared to those of the control mice (20.12±6.66 mmol/L versus 6.34±0.89 mmol/l, p<0.01). Ten-week 100 and 200 mg/kg TFF treatments decreased the blood glucose levels of the diabetic mice compared with the untreated diabetic mice (12.39±5.25 and 11.17±4.39 mmol/l versus 20.12±6.66 mmol/l, p<0.01).

**Table 1:** Effect of TFF on blood glucose levels of diabetic mice induced by STZ.

Group	Dose (mg/kg)	n	Blood glucose (mmol/l)	
			Initial	End
Control	-	9	6.81±1.01	6.34±0.89
DM	-	9	6.20±0.81	20.12±6.66*
DM+TFFI	50	9	6.67±0.59	19.28±3.66
DM+TFFII	100	9	6.81±0.47	12.39±5.25^#^
DM+TFFIII	200	9	6.41±0.49	11.17±4.39^#^

Data were expressed as mean ±standard deviation. *p<0.01 versus Control group; ^#^p<0.01versus DM group

### Total flavonoids from Flos Puerariae eased diabetic-induced bodyweight loss

**Figure 1:**
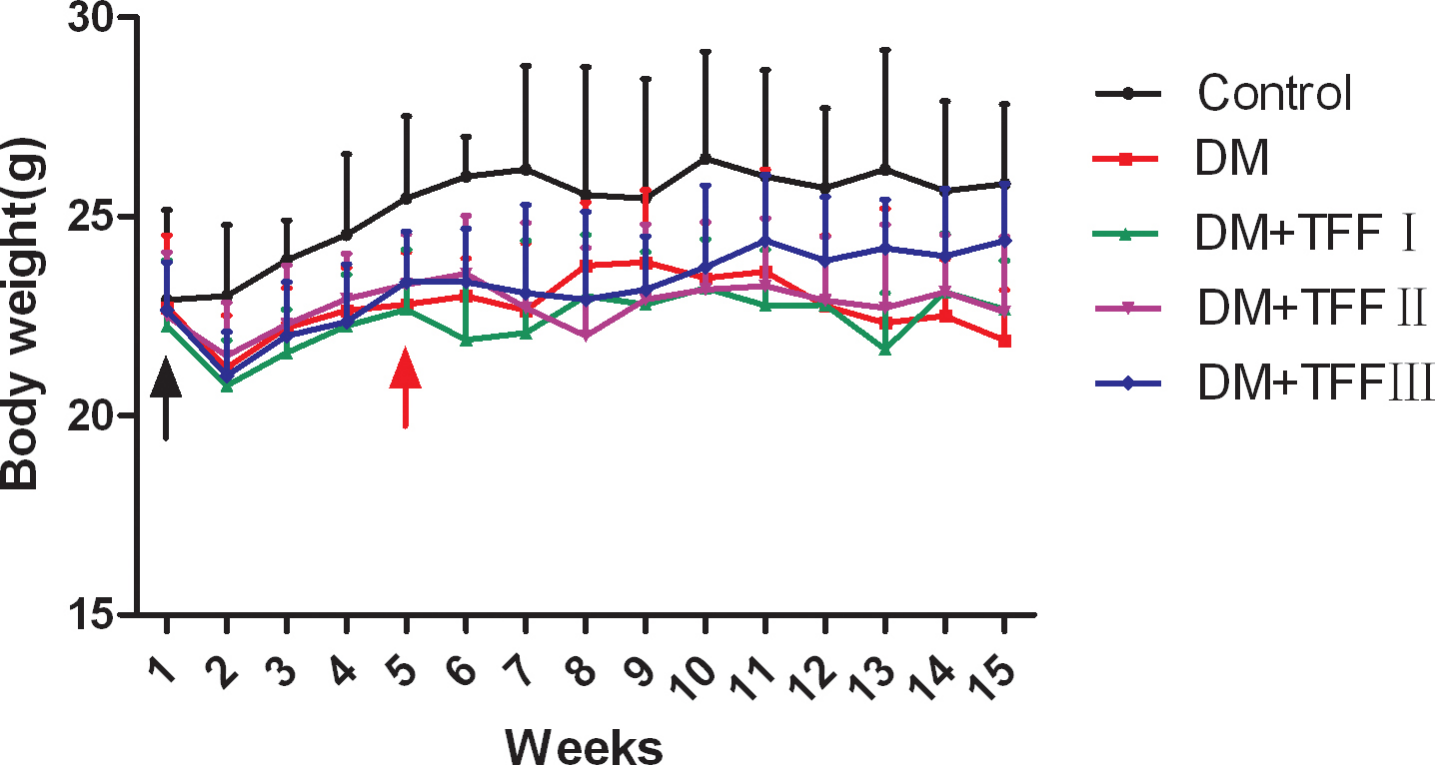
Effect of total flavonoids from Flos Puerariae (TFF) on the bodyweights of mice treated with streptozotocin (STZ) to induce diabetes. Data are expressed as the mean ± standard deviation; n=9 per group. The initial bodyweight in each group was similar (p>0.05). At 4 weeks after onset of the STZ treatment, the bodyweight of the diabetic model mice began to decrease. Then the mice in the drug intervention groups had intragastric TFF administration daily for 10 weeks. At the end of the experiment, the bodyweight of the DM group was less than that of control mice (p<0.01). The bodyweights of the 100 and 200 mg/kg TFF treatment mice (DM+TFF II and DM+TFF III) was greater than that of the DM group (p<0.05). The black arrow indicates intraperitoneal injecting of STZ. The red arrow indicates the beginning of TFF administration.

The line graph (Figure 1) shows the change in bodyweight for each group at each week of treatment. The initial bodyweight in each group was similar. The bodyweight of the control mice increased steadily over the 14-week course. At 4 weeks after the onset of the STZ treatment, the bodyweight of the diabetic model mice began to decrease. Subsequently, the bodyweight of the diabetic mice decreased 5.11% by 14 weeks. The bodyweight of the 50 and 100 mg/kg TFF-treated mice remained relatively stable and increased 1.89% and 2.67%, respectively, by 14 weeks. The bodyweight of the 200 mg/kg TFF-treated mice increased 8.22% by 14 weeks.

### Total flavonoids from Flos Puerariae prevented progressive damage of the diabetic mouse retina

**Figure 2:**
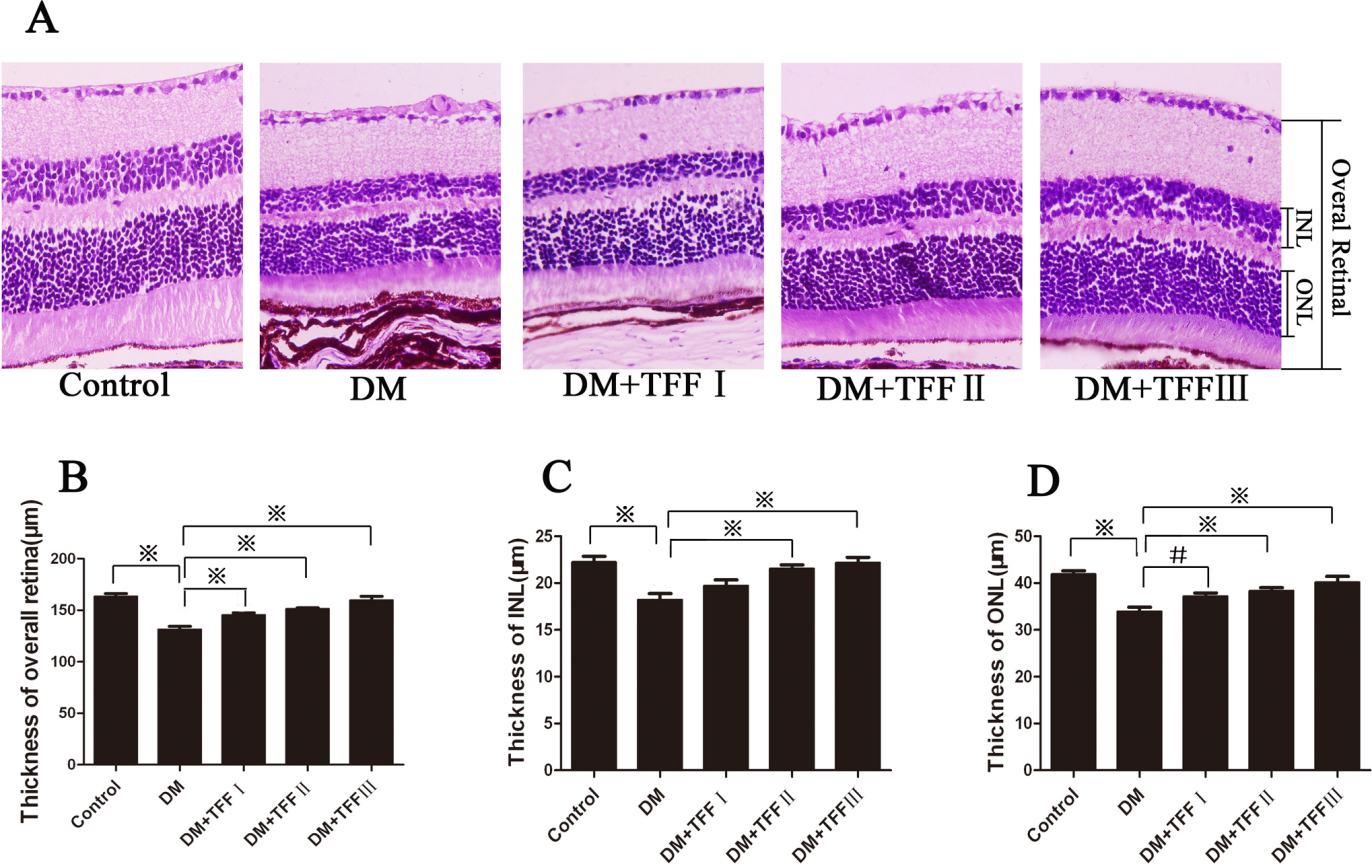
Effect of total flavonoids from Flos Puerariae (TFF) on the morphology of retinas in diabetic mice. **A**: The images were representatives the H&E–stained sections of retinas of the mice (Magnification: 400×). n=4 per group. INL: inner nuclear layer; ONL: outer nuclear layer. **B**-**D**: The thickness of the overall retina, the INL and the ONL were measured. Ten micrographs of each group were analyzed. Data are expressed as mean ±standard deviation. ^※^p<0.01; ^#^p<0.05.

A systematic morphology examination of the H&E-stained retinal sections (Figure 2) showed that the overall thickness of the retina in the diabetic mice was reduced by 19.58% compared with the control group (131.45±17.28 versus 163.45±13.58 μm, p<0.01). The thickness measurements of the INL and the ONL decreased by 18.17% and 18.99%, respectively, compared with the control group. In the diabetic mice treated with 50, 100, and 200 mg/kg TFF group, the retina appeared markedly more normal (Figure 2A), and the thickness of the overall retina increased compared with that of the diabetic group (145.46±17.05, 151.35±6.58 and 159.85±24.26 versus 131.45±17.28 μm, p<0.01). The thickness of the INL in the diabetic mice treated with 100 and 200 mg/kg TFF was significantly thicker than that in the diabetic group (21.84±1.99 and 22.18±3.22 versus 18.21±3.60 μm, p<0.01). The thickness of the ONL in the diabetic mice treated with 50, 100, and 200 mg/kg TFF was significantly greater than that in the diabetic group (37.21±4.49, 38.34±3.04 and 39.48±8.17 versus 33.95±4.92 μm, respectively, p<0.05).

### Total flavonoids from Flos Puerariae attenuated diabetes-induced ultrastructural changes

**Figure 3:**
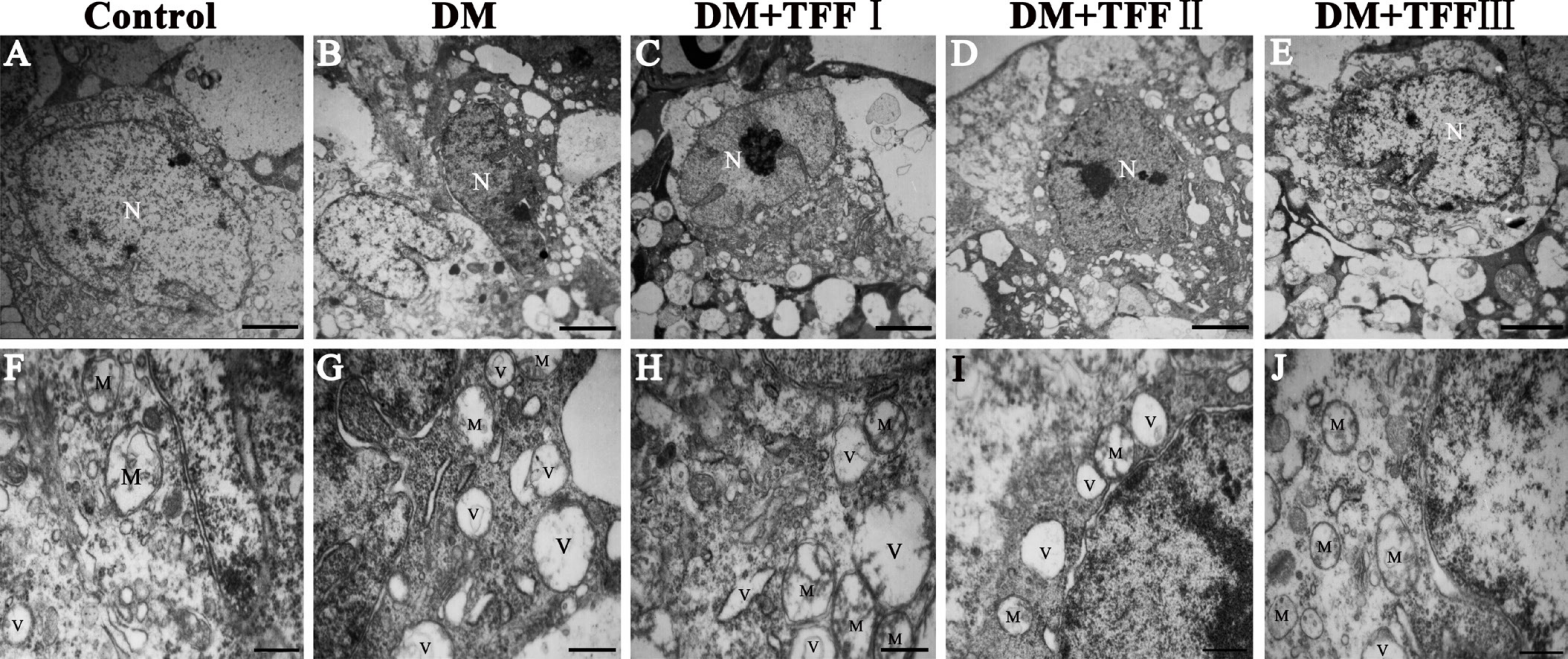
The protective effect of total flavonoids from Flos Puerariae (TFF) on the ultrastructure of the retinal ganglion cells. n=4 per group. Five electrophotographs of each group were analyzed. **A**–**E**: The representative images show the ultrastructural changes in the retinal ganglion cells (RGCs) of each group (Scale bar=3 μm). **F**–**J**: The enlarged views of **A**–**E** (Scale bar=1 μm). **A** and **F**: In the control group, the RGCs had uniformly normal-appearing nuclei (N) and mitochondria (M). **B** and **G**: In the DM group, vacuolation (V), chromatin margination, chromatin condensation, swelling of mitochondria, and loss of cristae were detected. **C** and **H**: In the 50 mg/kg TFF-treated group, there were still massive swollen mitochondria and chromatin condensation. **D** and **I**: In the 100 mg/kg TFF-treated group, mitochondria showed minor changes compared with the diabetic group, whereas chromatin margination and condensation exist. **E** and **J**: In the 200 mg/kg TFF-treated group, mitochondria and nucleus were basic normal and occasional evidence of apoptosis was detected. N: nucleus of the ganglion cell; M: mitochondria of the ganglion cell; V: vacuoles.

Retinal ultrathin sections were examined systematically with a transmission electron microscope to determine whether TFF affects apoptosis of the retinal ganglion cells (RGCs) and thickening of the retinal capillary basement movement (BM). The RGCs in the normal control mice had uniformly normal-appearing nuclei and mitochondria (Figure 3A,F), while vacuolation, chromatin margination, chromatin condensation, and swollen mitochondria were detected in the RGCs of the diabetic mice (Figure 3B,G). In the 50 mg/kg TFF-treated group, chromatin condensation and swollen mitochondria were detected (Figure 3C,H). The RGCs in the diabetic mice treated with 100 and 200 mg/kg TFF did not show obvious vacuolation, chromatin margination, chromatin condensation, or swollen mitochondria (Figure 3D,I,E,J).

**Figure 4:**
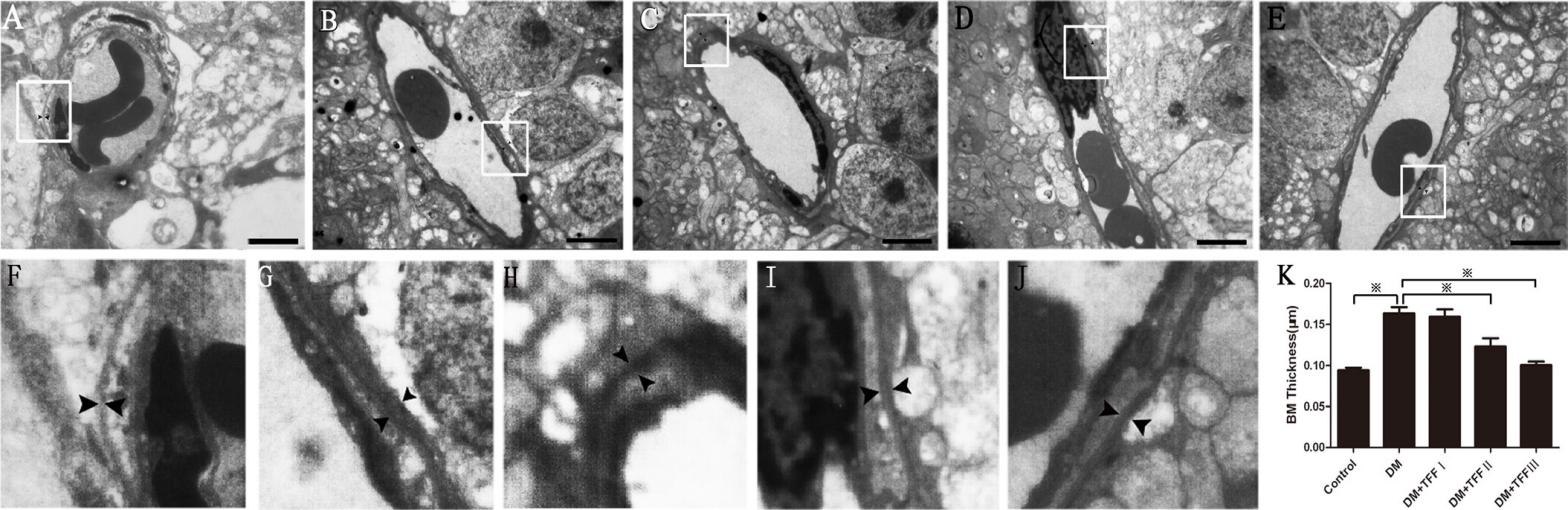
Treatment with total flavonoids from Flos Puerariae (TFF) attenuated the thickening of the retinal capillary basement membrane. n=4 per group. Eight electrophotographs of each group were analyzed. **A**–**E**: The electron micrograph images were representatives the transverse sections of retinal capillaries of the control (**A**), DM (**B**), 50 mg/kg TFF-treated (**C**), 100 mg/kg TFF-treated (**D**) and 200 mg/kg TFF-treated (**E**) group (Scale bar=4 μm). **F**-**J**:The enlarged views of **A**-**E** were showed. **K**: The BM thicknesses of retinal capillaries in different group were measured.. Data are expressed as mean ±standard deviation; n=4 per group. ^※^p<0.01.

An electron microscopy analysis of the retinal capillary (Figure 4) showed that the BM thickness of the retinal capillary was significantly increased in the diabetic mice compared to the normal control mice (0.16±0.05 versus 0.09±0.02 μm, p<0.05). After treatment with TFF, a significant decrease in retinal capillary BM thickness was observed in the retinas of diabetic mice compared to those of the diabetic model mice. The BM thickness of the retinal capillary in the 100 and 200 mg/kg TFF-treated group was significantly decreased compared with that of the diabetic model group (0.12±0.06 and 0.10±0.02 versus 0.16±0.05 μm, p<0.05).

### Total flavonoids from Flos Puerariae inhibited apoptosis of the neural cells in the diabetic mice retina

**Figure 5:**
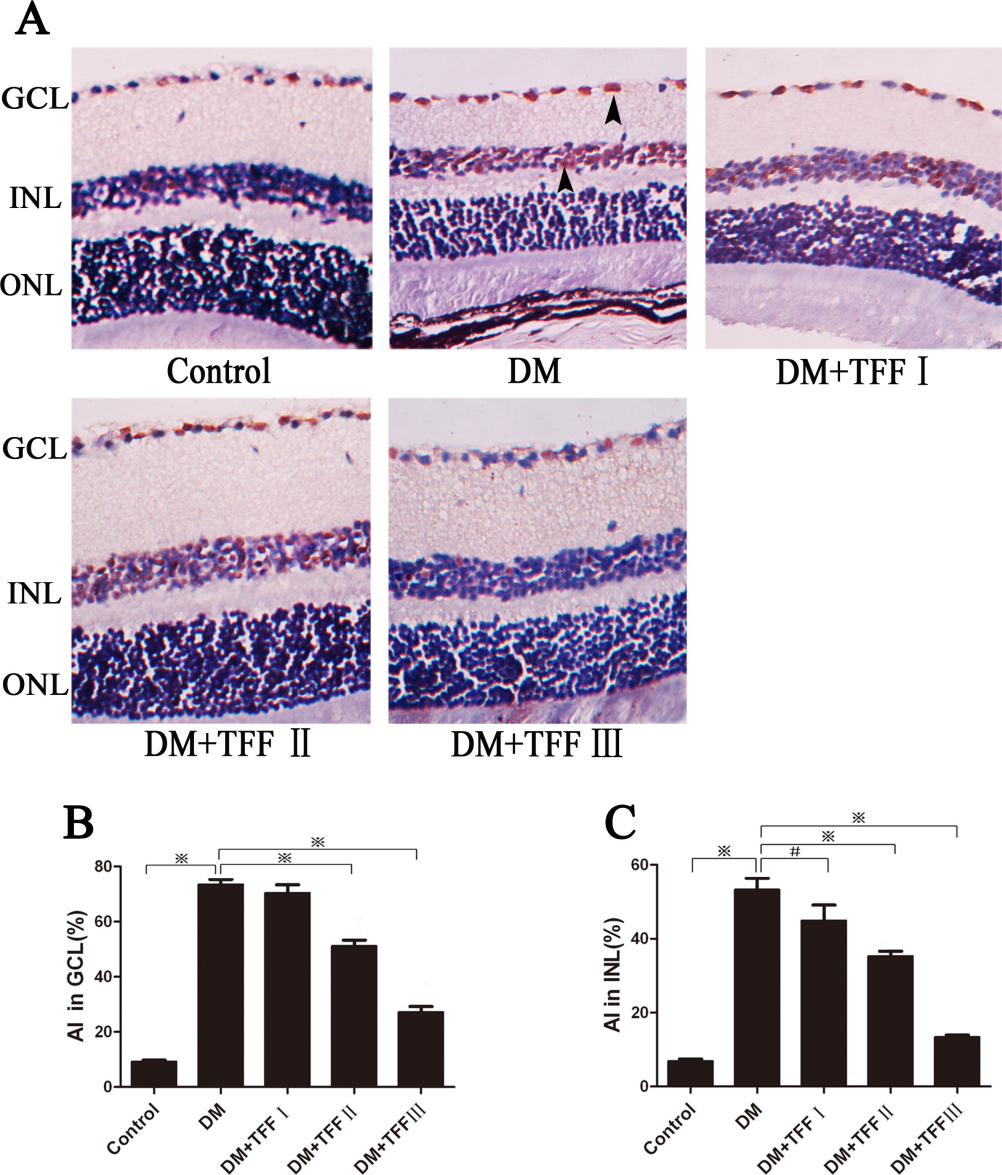
Treatment with total flavonoids from Flos Puerariae (TFF) attenuated apoptosis of neural cells in retinas of the diabetic mice. **A**: Representative images of TUNEL assay of the mice retinas of different groups (Magnification: 400×). n=4 per group. TUNEL-positive cells (*black arrows*) were distributed mainly in the ganglion cell layer (GCL) and the INL of the retinas. **B** and **C**: Apoptotic index in the GCL and the inner nuclear layer (INL) in different groups. Twelve micrographs of each group were analyzed. AI: apoptotic index; GCL: ganglion cell layer; INL: inner nuclear layer; Black arrows indicated TUNEL-positive cell. Data are expressed as mean ±standard deviation. ^#^p<0.05; ^※^p<0.01.

An examination of the retinas of diabetic mice showed more TUNEL-positive cells in the GCL and the INL than in the control mice (Figure 5A). The presence of TUNEL-positive cells was not detected in the outer nuclear layer. There were fewer TUNEL-positive cells in the GCL and the INL in the TFF-administered groups than in the diabetic model group, specifically in the 200 mg/kg TFF-treated group (Figure 5A). A quantitative analysis of the TUNEL-positive cells in the GCL and the INL of the retinas (Figure 5B,C) showed that the AI (apoptotic index, apoptotic nuclei/total nuclei ×100%) in the GCL and the INL of the diabetic mice increased eightfold and sevenfold, respectively, compared to that in the control mice. The 100 and 200 mg/kg TFF treatments significantly reduced the AI in the GCL compared with the diabetic group (51.18% and 27.16% versus 73.50%, p<0.01). The 50 mg/kg TFF treatment slightly reduced the AI in the INL compared with the diabetic group (44.95% versus 53.28%, p<0.05). The 100 and 200 mg/kg TFF treatments significantly reduced the AI in the INL compared with the diabetic group (35.28% and 13.35% versus 53.28%, p<0.01).

### Effect of total flavonoids from Flos Puerariae on Bax and Bcl-2 expression in retina

**Figure 6:**
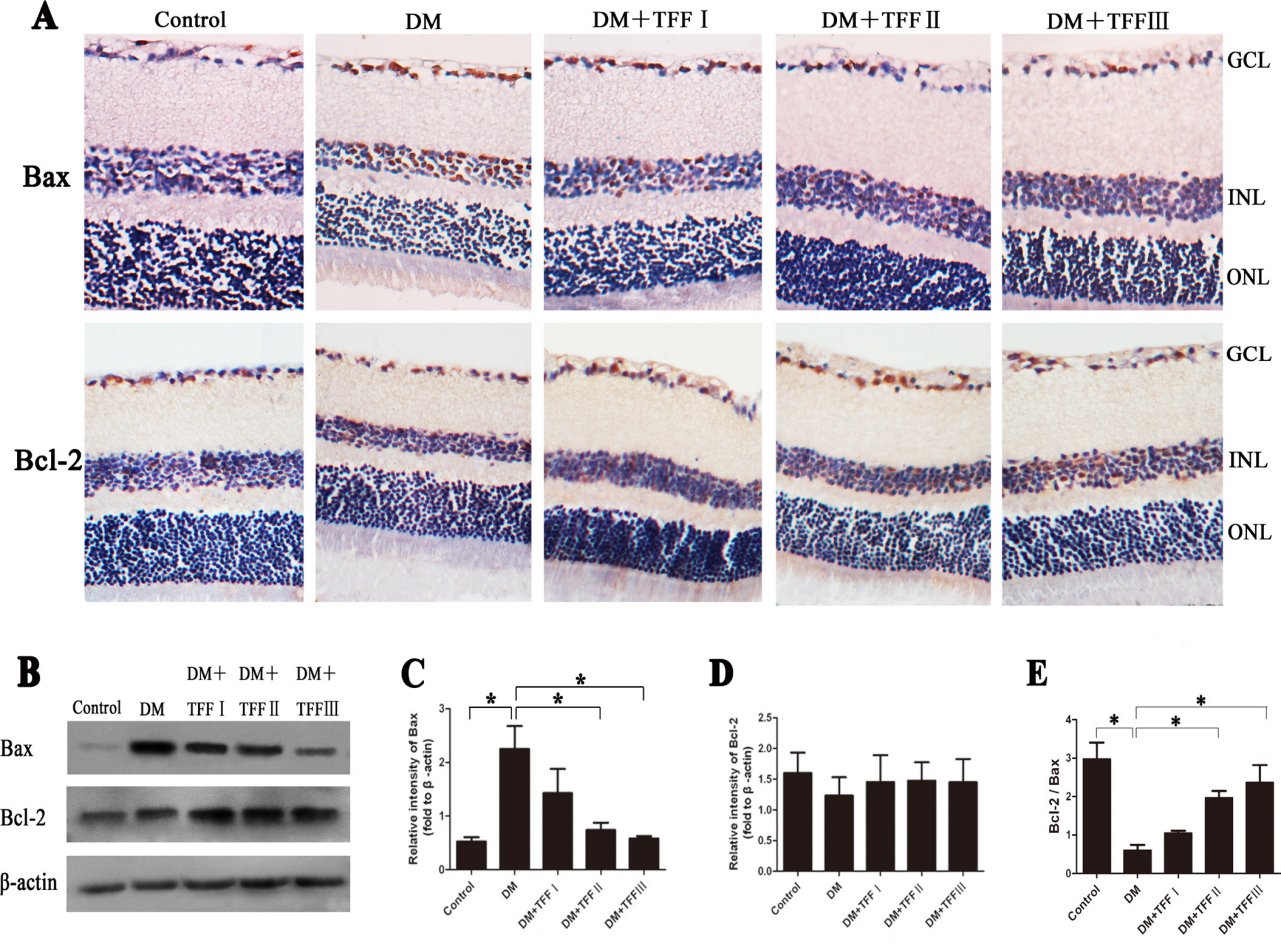
Effect of total flavonoids from Flos Puerariae (TFF) on Bax and Bcl-2 expression in retinas of the diabetic mice. **A**: The photomicrographs were representatives the retina sections immunohistochemistry stained with Bax and Bcl-2 antibody. n=4 per group. (Magnification: 400×). Bax and Bcl-2 expression were localized in the cytosol of cells mainly in ganglion cell layer (GCL) and inner nuclear layer (INL). **B**: The photographs were representatives the western blot of Bax and Bcl-2 protein. Five western blots per group were experimented. **C**: Relative intensities of Bax protein (fold to β-actin). **D**: Relative intensities of Bcl-2 protein (fold to β-actin). **E**: Ratio of the relative intensities of Bcl-2 to Bax (Bcl-2 */* Bax). Data are expressed as mean ±standard deviation. *p<0.01.

Bax and Bcl-2 expression, which is related to apoptosis, was observed using immunohistochemistry staining and western blotting (Figure 6). Immunohistochemistry staining showed that the Bax and Bcl-2 positive signal was localized in the cytosol of cells in the GCL and the INL. The immunohistochemistry and western blot analyses revealed that Bax expression significantly increased and Bcl-2 expression relatively decreased in the diabetic group compared with the control group. However, the 100 and 200 mg/kg TFF treatments decreased Bax expression compared with that of the diabetic group. The relative intensities of Bcl-2 (as normalized to the signal of β-actin) of each group showed no significant differences. As shown in Figure 6E, the expression ratio of Bcl-2 to Bax was significantly decreased in the diabetic mice retinas than in the control group (0.61±0.31 versus 2.98±0.95, p<0.01). The 100 and 200 mg/kg TFF treatments enhanced the ratio of Bcl-2 to Bax compared to the ratio in the diabetic group (1.97±0.39 and 2.37±1.01 versus 0.61±0.31, p<0.01). The ratio of Bcl-2 to Bax (Bcl-2/Bax) demonstrated the ability of TFF to inhibit apoptosis of the retina by upregulating Bcl-2 expression and downregulating Bax expression.

## Discussion

This study investigated the protective effects of TFF in preventing retinal neurocyte damage in STZ-induced diabetic mice. TFF reduced the blood glucose levels of diabetic mice and decreased diabetic-induced bodyweight loss in our study. We found that TFF markedly reduced retinal neuron injury related to diabetes, as demonstrated by an improvement in histological damage compared to the diabetic groups. We found that TFF prevented apoptosis of the retinal neurons by upregulating Bcl-2 expression and downregulating Bax expression in the diabetic mice, which suggests a possible protective mechanism of TFF in the retina.

Flos Puerariae has been reported to have a wide range of pharmacological effects. The isoflavonoid fraction of Flos Puerariae has been reported to prevent symptoms of excessive alcohol consumption (hangovers) [14] and to demonstrate antioxidant action [15]. Kakkalide has a liver protective effect [16], and kakkalide and tectoridin of Puerariae Flosan have an estrogenic effect [17]. Flos Puerariae extract is effective in treating metabolic diseases and displays hypolipidemic and hypoglycemic effects [18,19]. Flavones derived from *Radix Puerariae* exert inhibitory effects on bodyweight, abdominal fat content, and lipid levels in the liver [30] in which an increase in visceral fat is responsible for many of metabolic abnormalities, such as impaired glucose tolerance and insulin resistance, associated with abdominal obesity [31]. Kim et al. theorized that genistein may be a potential therapeutic agent for preventing and treating complications associated with diabetes mellitus, such as diabetic cataracts [32]. Aqueous extracts from Flos Puerariae protected against ethanol-induced apoptosis in the human neuroblastoma cell line SK-N-MC [20,21], and tectorigenin, extracted from Flos Puerariae, depressed t-BHP-induced-cellular cytotoxicity that may occur as a result of the inhibition of apoptosis [33]. In the early stage of this study, our colleagues demonstrated that the total flavones of Flos Puerariae (50, 100, and 200 mg/kg; intragastric administration) effectively protect against experimental liver injury induced by alcohol similar to previous research and that the total flavones of Flos Puerariae (50, 100, and 200 mg/kg/day; intragastric administration) could be a potential neuroprotective agent. We hypothesized that neuroprotective effects were achieved, at least in part, by the antioxygen free radical activities of Flos Puerariae, which suggest the potential of the total flavones of Flos Puerariae as an adjuvant therapy to conventional antihyperglycemic regimens for preventing and treating diabetic-associated cognitive decline. That article has not been published, and our study was performed based on those experimental results. We investigated the protective effect of the total flavones of Flos Puerariae on retinal neural cells in diabetic mice rather than fractionate it.

The pathogenesis of DR is complex and is not the consequence of a single mechanism. Neuronal cell death of the retina is a critical component of DR. This study clearly demonstrates the therapeutic potential of TFF in treating diabetic mice. This protective effect of TFF occurred in a dose-dependent manner. We demonstrated that the beneficial effects of TFF against the progression of diabetic retinopathy could be attributed to the hypoglycemic and antiapoptotic properties of TFF.

Studies have suggested that retinal neurodegeneration is a critical component of DR [3-7], which is typically characterized by a reduced number of RGCs, a thinned retina, and an increased number of apoptotic cells. A reduced number of RGCs, a thinned retina, and thinner inner and outer nuclear layers were detected in mice that had been diabetic for 10 weeks [4]. Barber et al. [34] identified a 22% and 14% decrease in the thickness of the inner plexiform and inner nuclear layers, respectively, of retinal sections from STZ diabetic rats after 7.5 months of STZ-induced diabetes. Oshitari et al. [3] reported that the number of TUNEL-positive cells in the ganglion cell layer of diabetic retinas was significantly increased compared to that in non-diabetic control rats. In this study, we noticed a significant decrease in retinal thickness and a distinct increase in TUNEL-positive cells in the retinas of diabetic mice. These TUNEL-positive cells were distributed predominantly in the GCL and the INL. The oxygen consumption of the neural cells in the GCL and the INL is abundant; thus, these cells are more sensitive to hypoxia and ischemia [35]. The ganglion cells in diabetic retinas express several proapoptotic molecules, suggesting that these cells are a vulnerable population [9]. These observations are consistent with previous studies. We found that TFF significantly improved morphological changes: The thickness of the entire retina and the inner and outer nuclear layers increased, and we detected decreased apoptosis in the GCL and the INL of diabetic mice. These improvements suggested that TFF can reduce the progression of experimental DR. Our data not only confirmed that apoptosis contributes to retinal neuronal death after the onset of diabetes but also demonstrated that TFF can prevent the apoptosis of retinal neurons. The apoptotic cells showed agglomeration and margination of chromatin [4], swelling, and vacuolation of mitochondria [36,37]. In the electron microscopy examination, we found obvious vacuolation, swollen mitochondria, margination of chromatin, and accidental apoptotic bodies in the GCL in the diabetic mice retinas, which further indicated that the cells of the ganglion cell layer were damaged via an apoptotic mechanism. There was no obvious change in the GCL of 100 and 200 mg/kg TFF-treated diabetic mice. We assume that TFF prevents the apoptosis of retinal ganglion cells by protecting the mitochondrial ultrastructure and maintaining cell integrity.

Studies on diabetic retinopathy have established a thickened vascular BM as one of the first structural abnormalities related to this condition. Kozak noted capillary BM thickening in the retinas of STZ-induced diabetic rats [38].Vascular BM serves as a substratum for cell attachment, provides a selective permeability barrier, and regulates cell survival [39]. Due to capillary BM thickening and apoptosis of pericytes, the integrity of the capillary was destroyed, and the blood–retinal barrier was damaged. These changes led to stenosis of the capillary and blood flow change, which promotes retinal ischemia [40]. The exclusive energy source for the retina is glucose. The glucose for retinal nerve activity is transported to the neurons predominantly in two ways: by the retinal artery and vein and by the choriocapillary [41,42]. According to the structural characteristics of the retina, a change in blood vessels may influence the energy supply for retinal neurons. Retinal capillary BM thickening may occur concurrently with neural cell apoptosis or perhaps earlier. This issue should be investigated in future research. In this study, we detected that the retinal capillary BM thickness of diabetic mice significantly increased compared to that of the normal control mice. TFF attenuated the capillary BM thickness of diabetic mice.

The mechanisms of apoptotic processes in retinal neural cells are unclear. Increased cellular apoptosis may be associated with Bax overexpression, which may be responsible, at least in part, for neuronal cell loss in the GCL of diabetic rats [3]. The Bcl-2 family members are involved in regulating activities concerned with the survival and death of neurons through apoptosis [43,44]. Bax, a mitochondrial membrane protein that mediates cell death, was increased in the retina of the diabetic subjects and may contribute to the development of vascular complications in diabetic retinopathy [45]. Several reports indicate that Bax is a critical factor in retinal neuronal cell apoptosis and a target for therapeutic gene strategy to protect damaged retinal neurons [46,47]. Bcl-2, an antiapoptotic member, inhibits cytochrome c release and the activity of proapoptotic members [48]. The intracellular Bcl-2 protein extended cell survival because it specifically blocked apoptotic cell death following various signals [43]. Proapoptotic Bax facilitates cytochrome c release and then activates caspases to induce apoptotic cell death [48,49]. The expression ratio of Bcl-2 to Bax is critical for determining the life span of cells [50]. In this study, a significant increase in Bax protein expression was found in the retina. The expression ratio of Bcl-2 to Bax clearly decreased, as it was implicated in apoptosis of retinal neural cells in previous studies. Administering 100 and 200 mg/kg TFF reduced the expression of the Bax protein and enhanced the ratio of Bcl-2 to Bax. The TFF-induced neuroprotection may have been mediated by normalizing the Bcl-2/Bax level, which attributed to the downregulation of Bax expression and the upregulation of Bcl-2 expression.

We found that TFF protected retinal neurons from undergoing apoptosis in experimental DR. This neuroprotective effect of TFF appeared to be related to the downregulation of Bax and the upregulation of Bcl-2. We concluded that TFF could have potential benefits in preventing the onset and progression of retinopathy in diabetic patients. The protective effects of TFF can be due to multiple beneficial effects on different retinal cells. Further investigation should be undertaken to clarify these beneficial effects.
